# Redox Control of IL-6-Mediated Dental Pulp Stem-Cell Differentiation on Alginate/Hydroxyapatite Biocomposites for Bone Ingrowth

**DOI:** 10.3390/nano9121656

**Published:** 2019-11-21

**Authors:** Silvia Sancilio, Eleonora Marsich, Helmut Schweikl, Amelia Cataldi, Marialucia Gallorini

**Affiliations:** 1 Department of Medicine and Ageing Sciences, G. d’ Annunzio University, Via dei Vestini 31, 66100 Chieti-Pescara, Italy; s.sancilio@unich.it; 2 Department of Medicine, Surgery and Health Sciences, Trieste University, Piazzale Europa 1, 34127 Trieste, Italy; emarsich@units.it; 3 Department of Conservative Dentistry and Periodontology, University Hospital Regensburg, University of Regensburg, D-93042 Regensburg, Germany; helmut.schweikl@ukr.de; 4 Department of Pharmacy, G. d’ Annunzio University, Via dei Vestini 31, 66100 Chieti-Pescara, Italy; cataldi@unich.it

**Keywords:** oxidative stress, Nrf2, IL-6, mineralization, dental pulp, scaffolds, alginate, hydroxyapatite, immunomodulation, nanomaterials

## Abstract

Composites and porous scaffolds produced with biodegradable natural polymers are very promising constructs which show high biocompatibility and suitable mechanical properties, with the possibility to be functionalized with growth factors involved in bone formation. For this purpose, alginate/hydroxyapatite (Alg/HAp) composite scaffolds using a novel production design were successfully developed and tested for their biocompatibility and osteoconductive properties in vitro. Redox homeostasis is crucial for dental pulp stem cell (DPSC) differentiation and mineralized matrix deposition, and interleukin-6 (IL-6) was found to be involved not only in immunomodulation but also in cell proliferation and differentiation. In the present study, we evaluated molecular pathways underlying the intracellular balance between redox homeostasis and extracellular matrix mineralization of DPSCs in the presence of composite scaffolds made of alginate and nano-hydroxyapatite (Alg/HAp). Prostaglandin-2 (PGE2) and IL-6 secretion was monitored by ELISA assays, and protein expression levels were quantified by Western blotting. This work aims to demonstrate a relationship between DPSC capacity to secrete a mineralized matrix in the presence of Alg/HAp scaffolds and their immunomodulatory properties. The variation of the molecular axis Nrf2 (nuclear factor erythroid 2-related factor 2)/PGE2/IL-6 suggests a tight intracellular balance between oxidative stress responses and DPSC differentiation in the presence of Alg/HAp scaffolds.

## 1. Introduction

Bone and dentin are nanostructured composites containing mineralized collagenous tissues owning mechanical properties uniquely adapted to their function [1]. During the early stages of odontogenesis, mesenchymal stem cells (MSCs) originating from the neural crest migrate toward the para-axial mesenchyme, taking up differentiation stimuli and releasing collagen fibers which form a mineralized matrix [2]. Adult stem cells are excellent resources for cell therapy and regenerative medicine. MSCs are adult pluripotent cells from the connective tissue, and they originate from the mesoderm [3]. Among these, dental pulp stem cells (DPSCs) were the first identified human MSCs capable of differentiating into odontoblastic, osteoblastic, neurogenic, adipogenic, myogenic, chondrogenic, and melanocytic lineages in vitro, and capable of forming a mineralized matrix similar to dentin or bone when transplanted into immunocompromised mice [4]. In addition to self-renewal and multilineage differentiation capacity, DPSCs also possess an immunomodulatory function, capable of secreting molecular signals and cytokines which can reduce inflammation and induce the regeneration of periodontal tissue [5].

Interleukin-6 (IL-6) acts as a pleiotropic cytokine in several cell processes such as immune regulation, hematopoiesis, and tissue regeneration in vivo. MSCs from the bone marrow (BM-MSCs) both secrete and respond to IL-6. Indeed, it was reported that autocrine/paracrine IL-6 is involved in the chondrogenic differentiation of BM-MSCs. However, the effect of IL-6 in the osteogenic differentiation of BM-MSCs is still controversial [6]. Xie and colleagues [7] found that BM-MSCs continuously secreted IL-6 under osteogenic induction. Evidence suggests that bone mass is affected by IL-6, which is capable of modulating osteocyte communication toward osteoclasts. However, the mechanism via which IL-6 enhances osteocyte-mediated osteoclastogenesis is unclear. It was shown that IL-6, TNF-α (tumor necrosis factor α), and IL-1β (interleukin-1β) contribute to bone remodeling in the early stages of fracture healing, and, after fracture surgery, they are involved in bone remodeling activation [8]. Additionally, bone resorption can be induced under IL-6 stimulation, and it can directly increase osteoclast formation in periodontal disease and rheumatoid arthritis. Wu et al. reported that bone remodeling is stimulated through IL-6 secretion in osteocyte and osteoclast precursors, speculating that it may be one of the fundamental mechanisms accelerating tooth movement by orthodontic surgery [9].

Annually, more than 900 million reconstructive surgeries are performed in response to bone and oral defects [10]. During the past few decades, several synthetic porous scaffolds were developed to replace autografts and allografts. Scaffolds are one of the key components in bone tissue-engineering strategies designed to regenerate damaged tissue [11]. Composite constructs made of biodegradable natural polymers were shown to be very promising, with excellent biocompatibility and suitable mechanical properties. For this purpose, we successfully developed alginate/hydroxyapatite (Alg/HAp) composite scaffolds using a novel production design [12]. The biocompatibility of these novel constructs was afterward evaluated in an in vitro cell system showing that DPSC osteogenic differentiation and mineralized matrix deposition are driven by the modulation of the antioxidant enzyme catalase. These findings suggested that DPSC osteogenic differentiation is tightly related to redox homeostasis, and that it is controlled by the activation of catalase which, as an enzymatic antioxidant, enhances cell survival in the presence of scaffolds [13]. It was well demonstrated that cell responses toward oxidative stress are driven by the transcription factor Nrf2 in different cell type [14,15]. Moreover, many diseases including bone disorders can be linked to oxidative stress. Changes in redox homeostasis are also related to the bone remodeling process, which implies constant bone regeneration through bone cell coordination. [16]. It was recently reported that a pharmacological induction of Nrf2 is involved in cytoprotection through a dose-dependent downregulation of pro-inflammatory cytokines, including prostaglandin-2 (PGE2) and IL-6 [17]. The metabolism of bone, as well as damage and disease processes, is strongly associated with the interplay between inflammation-related pathways (PGE2 in particular) and osteogenesis. Indeed, it was demonstrated that pro-inflammatory pathways of prostaglandins and bone morphogenic proteins are intertwined [18].

In this light, the present study was designed in order to evaluate the molecular pathways underlying the intracellular balance between redox homeostasis and extracellular matrix mineralization. Based on the above observations and reports, we investigated whether Alg/HAp scaffolds could be beneficial in the regulation of this molecular equilibrium in terms of protein expression related to antioxidant responses and cytokines secretion involved in osteogenic differentiation and inflammation.

## 2. Materials and Methods

### 2.1. Preparation and Characterization of Alginate/HAp Composites (Alg/HAp Scaffolds)

Alg/HAp composite scaffolds were prepared by mixing alginate 2% (*w*/*v*) and HAp 3% (*w*/*w*) in water using the calcium release method as previously described [12]. HAp powder was homogeneously dispersed into a stirred solution of alginate in water, followed by the addition of hydrolyzing d-gluconic acid δ-lactone 60 mM (GDL) to release calcium ions from HAp. Aliquots of this gelling solution were then cured in 24-well tissue culture plates (h = 18 mm, Ø = 16 mm, Costar, Cambridge, MA) for 24 h at room temperature to allow complete gelification. The hydrogels in the tissue-culture plate were then stepwise cooled by immersion in a liquid cryostat. Ethylene glycol in water (3:1) was used as a refrigerant fluid. Temperature was decreased stepwise from 20 °C to −20 °C by 5 °C steps with 30-min intervals for equilibration; the samples were then freeze-dried for 24 h to obtain isotropic network porous scaffolds [12].

### 2.2. Cell Culture of DPSCs on Alg/HAp Scaffolds

This project received the approval of the Local Ethical Committee of the University of Chieti-Pescara (approval number 1173, date of approval 31 March 2016) according to the Declaration of Helsinki. Obtained DPSCs from digested dental pulps were handled, cultivated, and characterized by their immunophenotype as previously described [19]. DPSCs were cultured and expanded in Minimun Essential Media (MEM)-α medium supplemented with 10% fetal bovine serum (FBS) and 1% penicillin/streptomycin (all purchased from EuroClone, Milan, Italy) up to the sixth passage.

### 2.3. Alg/HAp Scaffold Preparation for Cell Culture and Cell Seeding

Alg/HAp scaffolds underwent two cycles of sterilization under an ultraviolet (UV) light (15 W) for 1 h each, and they were rehydrated and conditioned in complete MEM-α overnight as previously reported [13]. After being expanded up to the sixth passage, DPSCs were trypsinized (trypsin/EDTA 1×, EuroClone, Milan, Italy) and collected by centrifugation (1200 rpm for 10 min at room temperature). DPSCs were then counted, and 5 × 10^4^ cells were resuspended in 130 μL of complete medium and afterward used for seeding drop by drop on each scaffold. Samples were immediately placed at 37 °C and 5% CO_2_ for 3 h to allow cell adhesion and interpolation onto/into scaffolds. Next, complete medium or differentiation medium (DM) was added to each sample (named in figures as Alg/HAp and Alg/HAp DM, respectively). Complete DM was supplemented as reported previously [13], with 100 μM ascorbic acid, 10 nM dexamethasone, 5 mM β-glycerol phosphate disodium salt pentahydrate (all purchased from Sigma Aldrich, MI, USA), and 1.8 mM potassium phosphate (Alfa Aesar Chemicals, Haverhill, MA, USA). DPSCs onto Alg/HAp scaffolds with or without DM were incubated for 1, 3, 7, 14, 21, and 28 days, and medium was refreshed every three days.

### 2.4. Protein Extraction and Quantification

After discarding cell supernatants, pellets from DPSC growth onto Alg/HAp scaffolds were obtained as previously reported [13]. In brief, scaffolds were dissolved in a sodium citrate buffer solution made from 50 mM sodium citrate tribasic dehydrate, 100 mM sodium chloride, and 10 mM sucrose (all purchased from Sigma Aldrich, MI, USA) and collected by centrifugation. After that, 0.5 mL of lysis buffer enriched with a protein inhibitor cocktail (PBS, 1% IGEPAL CA-630, 0.5% sodium deoxycholate, 0.1% SDS, 10 mg/mL PMSF, 1 mg/mL aprotinin, 100 mM sodium orthovanadate, and 50 μg/mL leupeptin, all purchased from Sigma-Aldrich, MI, USA) was added, and samples were kept on ice for 30 min. Then, pellets were resuspended in the enriched lysis buffer and kept on ice for an additional 30 min. Following centrifugation for 15 min at 20,000× *g*, the supernatant was collected as the whole-cell fraction. Protein concentration in the whole-cell lysate was determined using a bicinchoninic acid assay (QuantiPro™ BCA Assay Kit for 0.5–30 μg/mL protein, Sigma-Aldrich, Milan, Italy) following the manufacturer’s instructions. The absorbance at 562 nm was recorded in a spectrophotometer (Multiskan GO, Thermo Scientific, MA, USA), and the protein concentration (µg/mL) was determined by comparison to a standard curve using the Prism5 software (GraphPad, San Diego, CA, USA).

### 2.5. Immunoblot Analysis

Obtained quantified cell lysates (15 µg/sample) were electrophoresed and transferred to nitrocellulose membranes as reported in our previous work [13]. Membranes were afterward probed for mouse anti-β-actin monoclonal antibody (Sigma-Aldrich, St. Louis, MO, USA) (primary antibody dilution 1:10,000), rabbit anti- COX2, anti-PARP-1, and anti-Nrf2 polyclonal antibodies (all purchased from Santa Cruz Biotechnology, Dallas, TX, USA) (primary antibody dilutions 1:200, 1:500, and 1:750, respectively), as well as rabbit anti-p44/42 MAPK and anti-phosho-p44/42 MAPK (Erk 1/2 and p-Erk 1/2) monoclonal antibodies (all purchased from Cell Signaling Technology, Danvers, MA, USA) (primary antibody dilutions 1:1,000). Next, specific horseradish peroxidase-conjugated antibodies were added and immunoreactive bands were identified using the Enhanced Chemiluminescence (ECL) detection system (LiteAblot Extend Chemiluminescent substrate, EuroClone, Milan, Italy) and analyzed by densitometry through the ChemiDoc XRS System and the Quantity-One analysis software (Bio-Rad, Hercules, CA, USA). Protein band integrated optical intensities were normalized to that of β-actin (loading control).

### 2.6. Cytokine Assays

DPSCs (5 × 10^4^ cells) were resuspended in 130 μL of complete medium and seeded onto Alg/HAp scaffolds as previously described. Cell culture supernatants were collected after 1, 3, 7, 14, 21, and 28 days and analyzed for cytokine release. The amounts (pg/mL) of prostaglandin-2 (PGE2) and interleukin-6 (IL-6) were quantified using commercial ELISA kits (Enzo Life Sciences Inc, Lausen, Switzerland) following the manufacturer’s instructions. Absorbance was read at 405 nm for PGE-2 and at 450 nm for IL-6 by means of a spectrophotometer (Multiskan GO, Thermo Scientific, MA, USA). Cytokine concentration was determined by comparison to a standard curve using the Prism5 software (GraphPad, San Diego, CA, USA) according to the recommended calculation of results. Each of these values was normalized to the protein content (μg of protein/sample) measured by the BCA assay.

### 2.7. Statistical Analysis

Individual data from independent experiments were summarized as means ± standard error of the mean (SEM). Data were handled using Prism5 software (GraphPad, San Diego, CA, USA) using analysis of variance (one-way ANOVA). Significant differences between mean values were calculated using the *t*-test. Values of *p* < 0.05 were considered statistically significant.

## 3. Results

### 3.1. Cyclooxygenase-2 and PGE2 Modulation in DPSC Growth onto Alg/HAp Scaffolds

**Figure 1:**
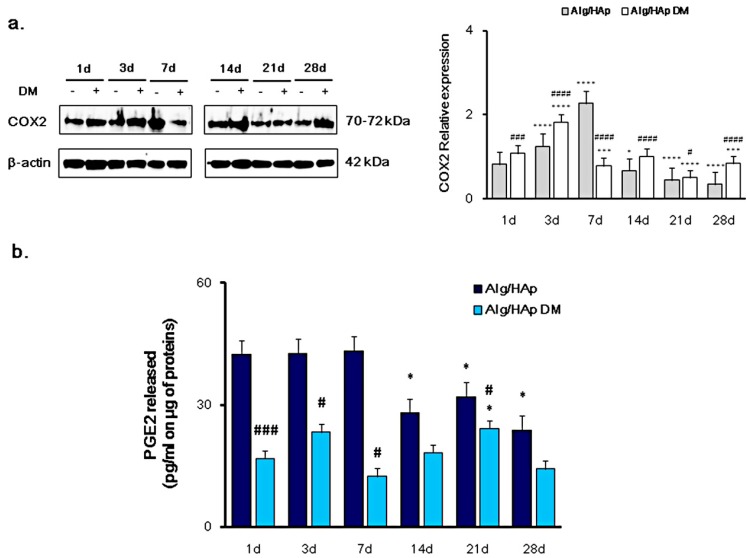
Cyclooxygenase-2/prostaglandin 2 COX2/PGE2 pathway modulation in dental pulp stem cells (DPSC) growth onto alginate/hydroxyapatite (Alg/HAp) scaffolds. (**a**) Typical results of COX2 protein expression by Western blotting of three independent experiments. β-actin was used as a protein content marker. The bar graph shows densitometric values expressed as the mean percentage ± Standard Error of the Mean (SEM) (*n* = 3) of integrated optical densities of protein bands normalized to β-actin. * *p* < 0.05 between Alg/HAp at 14 days and Alg/HAp at one day; *** *p* < 0.005 between Alg/HAp with or without differentiation medium (DM) at seven and 28 days and Alg/HAp at one day; **** *p* < 0.001 between Alg/HAp with or without DM at three days and Alg/HAp at one day; # *p* < 0.05 between Alg/HAp with and without DM at 14 days; ### *p* < 0.005 between Alg/HAp with and without DM at one day; #### *p* < 0.001 between Alg/HAp with and without DM at three, seven, and 28 days. (**b**) The bar graph displays the quantification of PGE2 released in pg/mL normalized to the protein content (μg/sample). Values are expressed as means ± SEM (*n* = 3). * *p* < 0.05 between Alg/HAp at 14, 21, and 28 days and Alg/HAp at one day; *p < 0.05 between Alg/HAp with DM at 21 days and Alg/HAp at one day; # *p* < 0.05 between Alg/HAp with and without DM at three, seven, and 21 days; ### *p* < 0.005 between Alg/HAp with and without DM at one day.

The expression of COX2 and PGE2 release was quantified as an evaluation of inflammation occurrence in DPSCs cultured in the presence of Alg/HAp scaffolds. When cells were cultivated without DM, levels of COX2 increase in a time-dependent manner from one day to three days (1.24 fold of β-actin) up to seven days, where a peak was assessed at 2.26-fold more than the marker control β-actin (Figure 1a). After that, COX2 was found to be downregulated, reaching the lowest value registered at 28 days (0.34-fold of β-actin). The presence of DM increased protein levels after one and three days of culture with respect to normal growth medium, but clearly moderated COX2 downregulation (0.77-fold of β-actin). After 14 and 28 days, there was only a slight protein expression increase with respect to DPSCs cultured without DM.

In accordance with COX2 expression, PGE2 release was dramatically enhanced after one, three, and seven days of culture from DPSC growth onto scaffolds without DM (Figure 1b), with no difference across the three experimental times (42.36 pg/mL, 42.67 pg/mL, and 43.29 pg/mL, respectively). As for 14, 21, and 28 days of culture, PGE2 secretion was significantly decreased, assessed at 27.99, 32.03, and 23.82 pg/mL, respectively. In the presence of differentiating agents, PGE2 released from DPSCs was substantially lower starting from early culture times. In detail, the pro-inflammatory cytokine concentration in samples with DM was 2.5-, 1.8-, and 3.5-fold lower compared to that with only MEM-α after one, three, and seven days of culture. After seven days, this ratio was slightly decreased due to the reduction of PGE2 released from DPSCs grown onto scaffolds without DM, but it was still registered (Figure 1b).

### 3.2. Erk 1/2 Phosphorylation and PARP-1 Cleavage in the DPSC/Scaffold Model

**Figure 2:**
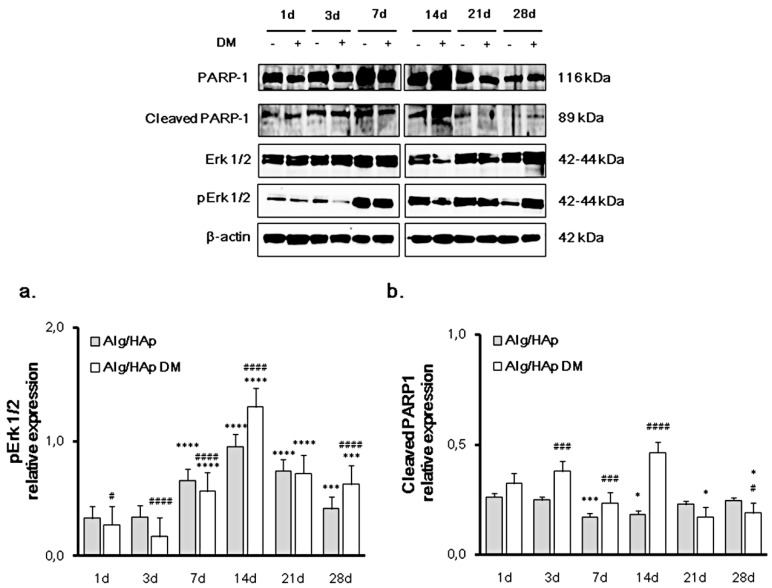
Extracellular signal-regulated kinases (Erk 1/2) phosphorylation and cleavage of Poly [ADP-ribose] polymerase-1 (PARP-1) and in DPSCs growth onto Alg/HAp scaffolds. Typical results of Erk 1/2 and PARP-1 protein expression by Western blotting of three independent experiments. β-actin was used as a protein content marker. The bar graphs show densitometric values expressed as the mean percentage ± SEM (*n* = 3) of integrated optical densities of protein bands normalized on β-actin. (**a**) *** *p* < 0.005 between Alg/HAp at 28 days with or without DM and Alg/HAp at one day; **** *p* < 0.0005 between Alg/HAp at seven, 14, and 21 days with or without DM and Alg/HAp at one day; # *p* < 0.05 between Alg/HAp with and without DM at one day; #### *p* < 0.0005 between Alg/HAp with and without DM at three, seven, 14, and 28 days. (**b**) * *p* < 0.05 between Alg/HAp at 14 days and Alg/HAp at one day; * *p* < 0.05 between Alg/HAp with DM at 21 and 28 days and Alg/HAp at one day; *** *p* < 0.005 between Alg/HAp at seven days and Alg/HAp at one day; # *p* < 0.05 between Alg/HAp with and without DM at 28 days; ### *p* < 0.005 between Alg/HAp with and without DM at three and seven days; #### *p* < 0.001 between Alg/HAp with and without DM at 14 days.

In order to investigate whether the modulation of pro-inflammatory proteins activates the regulation of repairing pathways related to inflammation and cell survival, Erk 1/2 activation and PARP-1 expression or cleavage were investigated in the DPSC/Alg/HAp scaffold model. As for Erk 1/2 activation, the increase in protein phosphorylation was clearly time-dependent up to 14 days, and it was enhanced by DM (Figure 2a). Circumstantially, after seven and 14 days of culture onto Alg/HAp scaffolds, levels of p-Erk 1/2 were respectively 0.66- and 0.96-fold of the total protein without DM and 0.56- and 1.30-fold in the presence of DM. After that, there was a fall in the expression of activated Erk 1/2, with values of phosphorylated protein at 28 days assessed at 0.41-fold without DM and 0.63-fold in the presence of differentiation agents (Figure 2a).

In general, the amount of cleaved PARP-1 was not higher than 0.5-fold of PARP-1, due to the large amount of the full-length protein (Figure 2b). Outstandingly, the presence of differentiation agents in culture enhanced PARP-1 cleavage mainly at 14 days (0.47-fold of the full length). After 21 and 28 days, levels of cleavage were comparable or even lower than the first day of culture.

### 3.3. Nrf2 Early Activation in DPSCs in the Presence of Alg/HAp Scaffolds

**Figure 3:**
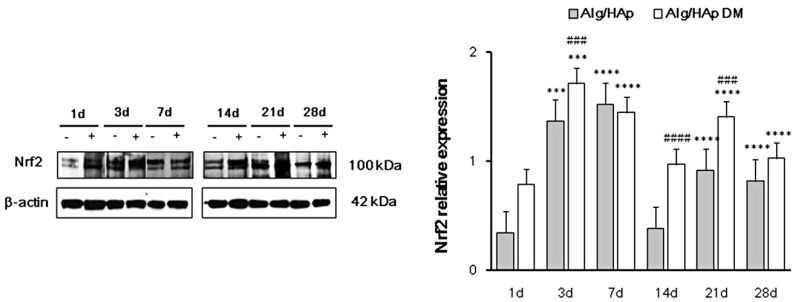
Nuclear factor erythroid 2-related factor 2 (Nrf2) expression levels in DPSC growth onto Alg/HAp scaffolds. Typical results of Nrf2 protein expression by Western blotting of three independent experiments. β-actin was used as a protein content marker. The bar graph shows densitometric values expressed as the mean percentage ± SEM (*n* = 3) of integrated optical densities of protein bands normalized on β-actin. *** *p* < 0.005 between Alg/HAp at three days and Alg/HAp at one day; *** *p* < 0.005 between Alg/HAp with DM at three days and Alg/HAp at one day; **** *p* < 0.001 between Alg/HAp at seven, 21, and 28 days and Alg/HAp at one day; **** *p* < 0.001 between Alg/HAp with DM at seven, 21, and 28 days and Alg/HAp at one day; ### *p* < 0.005 between Alg/HAp with and without DM at three and 21 days; #### *p* < 0.001 between Alg/HAp with and without DM at 14 days.

Since changes in redox homeostasis are linked to bone remodeling [16] and Nrf2 is a master regulator of cell responses toward oxidative stress [14], expression levels of this transcription factor were analyzed and quantified by immunoblotting. Nrf2 was only slightly expressed after one day in DPSC seeded on scaffolds (0.34-fold), but levels of expression were quite enhanced by differentiation agents at the same experimental time (0.79-fold) (Figure 3). After three days of culture, Nrf2 levels were even more increased, sensitive to the presence of differentiation medium (1.37- and 1.71-fold of β-actin, respectively). Furthermore, Nrf2 expression was significantly increased after seven days of culture, mainly when DPSCs were grown in the presence of normal medium (1.52- and 1.44-fold of β-actin). Compared to these levels, a dramatic fall was registered after 14 days of culture without DM (0.38-fold of β-actin), while Nrf2 expression levels remained quite high in the presence of differentiation agents (0.97-fold). Then, after 21 days of culture, Nrf2 levels were enhanced mainly by DM (1.40-fold of β-actin). Finally, Nrf2 expression was comparable to that at 21 days when DPSCs were grown on scaffolds for 28 days, with the protein relative expression assessed at 0.81-fold.

### 3.4. Influence of Alg/HAp Scaffolds on IL-6 Released from DPSCs

**Figure 4:**
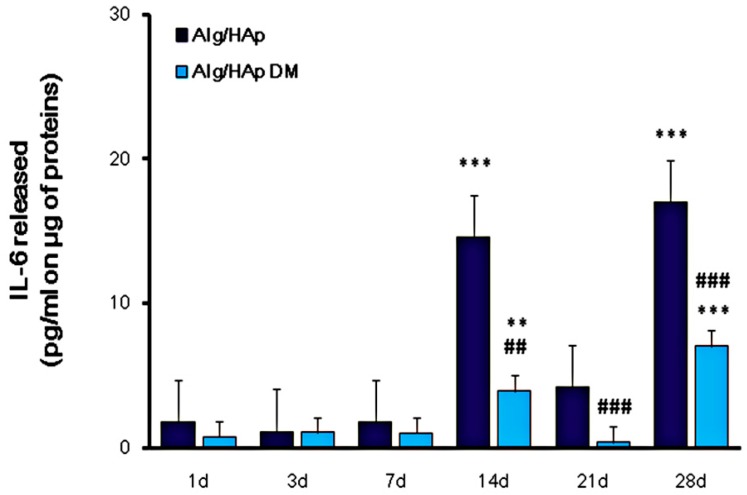
Interleukin-6 (IL-6) released from DPSC growth onto Alg/HAp scaffolds. The bar graph displays the quantification of IL-6 released in pg/mL normalized to the protein content (μg/sample). Values are expressed as means ± SEM (*n* = 3). ** *p* < 0.01 between Alg/HAp at three days and Alg/HAp at one day; ** *p* < 0.01 between Alg/HAp with DM at three and 14 days and Alg/HAp at one day; *** *p* < 0.005 between Alg/HAp at 14 and 28 days and Alg/HAp at one day; *** *p* < 0.005 between Alg/HAp with DM at 28 days and Alg/HAp at one day; ## *p* < 0.01 between Alg/HAp with and without DM at 14 days; ### *p* < 0.005 between Alg/HAp with and without DM at 28 days.

To verify whether IL-6 secretion could be involved in DPSC osteogenic differentiation, we investigated the modulation of the cytokine secretion in the Alg/HAp/DPSC model by means of an ELISA assay. Initially and up to seven days of culture, amounts of IL-6 secreted in supernatants were hardly detectable in the DPSC/scaffold model, and they were even significantly decreased after three days with respect to one day of culture (Figure 4). Moreover, the amount of cytokine released was independent of the presence of DM. After 14 days of culture, the IL-6 concentration dramatically raised, secreted 8.5-fold more than that after one day of culture from DPSCs grown onto Alg/HAp scaffolds without DM (14.57 pg/mL and 1.77 pg/mL, respectively). In parallel, IL-6 released in the presence of DM was lower, assessed at 3.94 pg/mL at the same experimental time. After 21 days of culture, IL-6 amounts were significantly decreased, mainly in DPSCs grown in the presence of DM (0.41 pg/mL). Contrariwise and comparably to 14 days of culture, IL-6 was remarkably released after 28 days with and without DM (7.05 pg/mL and 17.03 pg/mL, respectively). Notably IL-6 concentration in cell supernatants in the absence of DM was doubled compared to DPSCs cultured onto scaffolds and DM (Figure 4).

## 4. Discussion

Conservative therapies based on filling materials, fixed dental bridges, or removable dentures and dental implants are used to replace tooth structure or missing teeth [20]. Biomechanical overload, osseointegration failure, infections, and inflammation are common complications of surgical procedures [21], as well as failed cell adhesion on the material surface due to bacterial interference or ineffective coating [22]. Transforming growth factor beta (TGF-β), interleukin-6 (IL-6), interleukin-10 (IL-10), hepatocyte growth factor (HGF), prostaglandin E2 (PGE2), and human leukocyte antigen G (HLA-G) are secreted by DPSCs as anti-inflammatory cytokines. We previously reported that the inducible cyclooxygenase (COX2) is involved in the modulation of inflammation events in the presence of collagen membranes for periodontal bone and peri-implant defect treatments [23], and that PGE2 production can be involved in many differentiation processes, including osteogenic and angiogenic differentiation in the presence of biomaterials [19,24]. However, the anti-inflammatory effects of DPSCs during biomaterial integration after implantation are still poorly understood, and there is growing interest in studying the immunomodulatory effectiveness of DPSCs [25]. In the Alg/HAp scaffold/DPSC model, the expression of COX2 gradually and significantly increased up to seven days of culture mainly when only growth medium was present, and this trend was in alignment with PGE2 secretion (Figure 1). After seven days, inflammation was dramatically reduced, as well as cytokine production, but not to the same extent. This finding was consistent with our previous results on biocompatibility of Alg/HAp scaffolds, where the amount of LDH enzyme secreted after one day was 11-fold higher than that released from DPSCs alone and, after this peak, the percentage of LDH released gradually decreased up to 28 days of culture [13]. In the present work we, therefore, investigated the molecular mechanisms underlying the promising biocompatibility data on Alg/HAp scaffolds and whether they are interconnected with DPSC commitment to the odontogenic lineage.

Mitogen-activated protein kinases (MAPKs) are involved in physiological responses such as cell proliferation and differentiation. It was observed that biocomposite scaffolds containing diosmin exerted an osteostimulatory effect driven by the integrin/FAK/Erk signaling pathway in mouse mesenchymal stem cells [26]. In the presence of Alg/HAp scaffolds, expression levels of phosphorylated Erk 1/2 revealed a peak after 14 days, indicating that cells may avoid inflammation through this molecular pathway. In detail, Erk 1/2 activation was more evident in the presence of DM, while COX2 expression and PGE2 secretion were more increased with scaffolds in normal growth medium. It is plausible to assume that signaling through the Erk pathway is enhanced in the presence of DM, confirming our previous LDH data when the LDH percentage was higher with respect to DPSCs and scaffold alone at 14 days of culture [13].

Poly (ADP-ribose) polymerase 1 (PARP11) participates in processes such as transcription and DNA repair through the regulation of chromatin structure. Recently, it was revealed that PARP-1 activation is driven by other mechanisms not involving its binding to DNA strand breaks. PARP-1 becomes activated downstream in the MAP kinase phosphorylation cascade by binding to phosphorylated Erk 1/2 [27]. In our experimental model, PARP-1 was increased after 14 days of culture in the presence of Alg/HAp scaffolds and DM in alignment with the phosphorylation of Erk 1/2, and this observation was concurrent with the decrease in COX2 expression levels after seven days of culture. Lin and colleagues [28] identified PARP-1 as one of the transcription factors binding to the repressor element in the promoter region of COX2. It is plausible to assume that, in our experimental model, the presence of Alg/HAp biocomposites decreased inflammation mediator release and, in parallel, increased the expression of proteins related to cell survival and proliferation. This process enhanced cell escape from necrotic cell death and DPSC commitment toward the odontogenic lineage and mineralization, as demonstrated in our previous study [13].

In a clinical situation, cytokine release is the result of the inflammation of the dental pulp tissue after surgical practice, and this cell response triggers the synthesis of regulatory proteins activated by stressors. Adaptive cell responses to inflammation are associated with oxidative and nitrosative stress, and it was largely demonstrated that redox homeostasis perturbance in the presence of biomaterials is driven by the activation of the redox-sensitive transcription factor Nrf2 and Nrf2-related proteins [29]. We previously reported a relationship between cell escape from necrotic cell death and the increase in catalase activity in the presence of Alg/HAp scaffolds [13]. Since a redox homeostasis is crucial for osteogenic commitment and differentiation [30] and Nrf2 is a fine regulator of the antioxidant enzyme catalase, we monitored here expression levels of this transcription regulator.

In the Alg/HAp scaffold/DPSC system, there was a peak in the expression of Nrf2 after early exposure periods to scaffolds, with the expression doubled with respect to one day of culture. After seven days, this expression was maintained high and significant with no difference between DM and growth medium. This could be related to our previous data on catalase activity when, at seven days of culture onto scaffolds, the absolute highest value of catalase activity was registered [13]. In the present study, there was a decrease in Nrf2 expression after 14 days of culture, mainly in the normal growth medium. After that, Nrf2 expression showed a significant increase in the presence of scaffolds and DM, and it decreased after 28 days. We previously reported that bone sialoprotein II (BSP II) is notably expressed after 21 and 28 days, and we observed two positive pulses with regard to gene expression of *RUNX2* after 21 days and *SP7* after 28 days when scaffolds and DM were present, suggesting differentiation of DPSCs [13]. There is growing evidence that the overexpression of Nrf2 is a possible target protein involved in stem-cell marker expression and in the enhancement of osteoblastic differentiation [31]. Alg/HAp scaffolds are, therefore, suitable materials for bone ingrowth not only because of their physical properties and composition resembling the native bone structure, but also because they facilitate the expression of key molecules involved in redox homeostasis and differentiation, decreasing cytokine release and inflammation.

Among cytokines, IL-6 with its receptor plays an important role in tissue regeneration in vivo, especially hard tissue metabolism [7]. In our experimental model, IL-6 secretion was not consistent up to seven days of culture in both experimental situations, i.e., in the presence of Alg/HAp scaffolds with or without DM (Figure 4). After that, there was a dramatic peak in the presence of normal growth medium, whereas, in the presence of DM, the increase was lower but still consistent at 14 days. This was in alignment with our previous data on gene expression of differentiation markers. Indeed, a significant positive peak of *BMP2* expression levels was shown in our previous investigation after seven days of culture without DM [13]. Expression of *BMP2* is recognized to stimulate osteogenic commitment of DPSCs, considered as the most osteogenic bone morphogenetic protein [32]. It is, therefore, plausible to speculate that the peak of IL-6 is triggered by BMP2 expression as a stimulus for DPSC differentiation. In addition, it was reported that MSCs both secrete and respond to IL-6 [7], and it was reported that the modulation of IL-6 is important in the extension of the osteoblastic stage of primary human osteoblasts [33]. In this view, it is not surprising that, after this peak, there was a significant fall of IL-6 secretion, possibly as a consequence of a negative feedback mechanism. In parallel, an increase of protein levels related to the molecular pathway involved in inflammation was likely because of the increase in Nrf2 after 21 days of culture. Likewise, we showed a decrease in Nrf2 expression levels in parallel with an increase in IL-6 secretion at 28 days.

## 5. Conclusions

Taken together, our results partly present the molecular mechanisms underlying the biological processes of DPSC differentiation in parallel with the escape from oxidative stress. In the present work, we reported a relationship between DPSC capacity to secrete a mineralized matrix in the presence of Alg/HAp scaffolds and their immunomodulatory properties. The modulation of the Nrf2/PGE2/IL-6 molecular axis suggests a tight intracellular balance between oxidative stress responses and DPSC differentiation in the presence of Alg/HAp scaffolds. The present study lays the groundwork for further molecular investigations using pharmacological modulators and/or knock-down techniques, in an effort to further demonstrate the interplay between pro-inflammatory pathways and bone remodeling in the presence of nanomaterials designed for tissue-engineering purposes.
